# Ultrasonography in disaster medicine: a narrative review

**DOI:** 10.1186/s13244-026-02420-3

**Published:** 2026-10-13

**Authors:** Félix Amiot, Karim Tazarourte, Guillaume Gorincour, Axel Benhamed

**Affiliations:** 1 Emergency Department–SAMU50, Center Hospitalier Mémorial Saint-Lô, Saint-Lô, France; 2 SAMU69–Urgence CHU Edouard Herriot, RESHAPE U1290 Université Lyon1, Lyon, France; 3 Mediterranean Institute for Medical Imaging in Gynecology, Obstetrics and Children, Marseille, France; 4 Emergency Department–Research Center, CHU de Québec-Université Laval, Québec, QC Canada

**Keywords:** Ultrasonography, Point-of-care systems, Disaster medicine, Triage, Telemedicine

## Abstract

**Abstract:**

This narrative review aimed to critically appraise the diagnostic accuracy, clinical impact, and operational feasibility of ultraportable point-of-care ultrasonography (POCUS) across the four domains of disaster medicine. A literature search was conducted following the SANRA and PRISMA-trAIce guidelines. Records were identified through an AI-assisted literature-discovery tool (the Undermind Deep-Search platform, interrogating the Semantic Scholar corpus) run on 15 June 2025, rather than through an independent structured multi-database search. Candidate references were screened manually against pre-specified eligibility criteria by a single reviewer, with the search parameters independently verified by the senior author. Of 71 records identified, 63 met the inclusion criteria. The included studies comprised 32 original studies, 10 systematic or scoping reviews, 20 narrative or operational papers, and one guideline. In CBRN scenarios, lung ultrasound demonstrated sensitivities of 90–97% for COVID-19 pneumonia versus 60–69% for chest radiography while minimizing healthcare worker exposure. For blast and ballistic injuries, FAST showed a pooled sensitivity of 92.1% (95% CI: 87.8–95.6) and specificity of 98.7% (95% CI: 96.0–99.9) for intra-abdominal hemorrhage. In natural disasters, ultrasound altered management in up to 44% of patients. Post-disaster applications included vascular access, tube confirmation, and complication monitoring. Ultraportable POCUS demonstrates diagnostic utility and operational feasibility across multiple disaster domains. However, evidence quality varies significantly, and standardized protocols remain lacking. Prospective studies linking ultrasound deployment to improved patient outcomes are needed.

**Key Points:**

**Question** When disasters disable conventional imaging, how does ultraportable point-of-care ultrasonography perform for diagnosis, triage, and procedural guidance across the four domains of disaster medicine?

**Findings** Across 63 studies, FAST reached 92.1% pooled sensitivity for hemoperitoneum, lung ultrasound 90–97% versus 60–69% for radiography; management changed in up to 44%.

**Critical relevance statement** This review critically appraises ultraportable ultrasound across all four disaster domains, showing that radiation-free bedside imaging accelerates triage, conserves scarce CT capacity, and extends radiological expertise through tele-guidance, while exposing an evidence base still lacking prospective outcome data.

**Graphical Abstract:**

**Figure:**
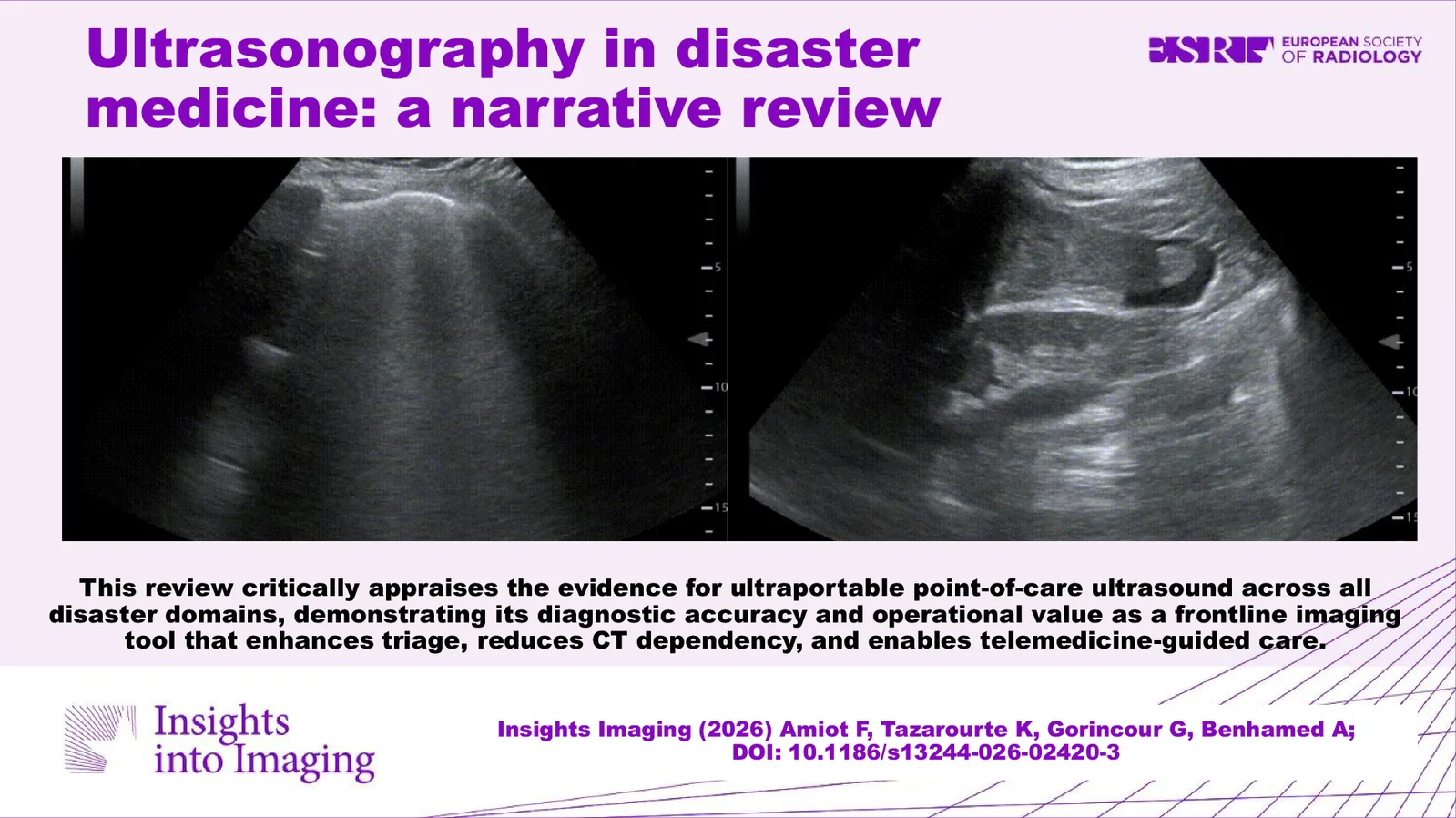

## Introduction

Ultrasonography has become an essential tool in emergency medicine, providing efficient, rapid, noninvasive, and real-time imaging at the bedside. It is now extensively integrated into emergency medicine practice, with point-of-care ultrasound (POCUS) enhancing diagnostic accuracy, facilitating time-critical decision-making, and guiding interventions in acute settings [1, 2]. The pivotal role of ultrasonography in prehospital and in-hospital emergency care is widely recognized by international guidelines, including those from the American College of Emergency Physicians and European emergency medicine societies [1–3]. Technological advances have transformed medical ultrasound devices, particularly with the emergence of handheld and ultraportable scanners [4]. These compact ultrasound scanners offer high-quality imaging while being lightweight and highly portable [5, 6]. Originally designed to extend medical imaging access to patients regardless of their location, these devices have since been adopted in military and austere medical settings [7]. In remote environments where resources are limited and patient transport may be hampered or delayed, handheld ultrasound devices enable immediate bedside imaging and timely clinical decision-making [8]. These devices contribute to improving diagnostic performance and patient management [9].

Disaster medicine presents a unique operational context characterized by extreme conditions, resource scarcity, and an imperative for rapid triage and intervention. During disaster response, conventional diagnostic imaging, namely CT and radiography, requiring fixed installations and substantial electrical infrastructure, becomes rapidly unavailable owing to facility damage, power disruption, or overwhelming patient volume. Traditional ultrasound machines, constrained by their bulk and prolonged boot-up times, have historically been of limited use in these settings [6]. The advent of ultraportable ultrasound devices, with rapid activation and robust imaging capabilities, now allows their practical integration into disaster response [10]. These innovations allow ultrasound to be deployed anywhere, opening new opportunities for prehospital disaster care that were previously impractical. Furthermore, field teams can now perform on-scene sonographic assessments, real-time monitoring of patients’ status, and ultrasound-guided interventions in austere conditions. Critically, ultraportable ultrasound minimizes healthcare worker exposure to infectious or toxic contamination by obviating the need for patient transport to distant imaging facilities and reducing the number of personnel required for assessment.

Notably, previous syntheses have typically examined ultrasonography either through the lens of military medicine or within the broader context of austere and remote care, creating the need for an integrated disaster medicine perspective that bridges civilian and military settings [8, 9, 11]. This narrative review aims to critically appraise the diagnostic accuracy, clinical impact, and operational feasibility of ultraportable point-of-care ultrasonography across the four domains of disaster medicine: CBRN hazards, explosive and ballistic trauma, natural disasters, and prolonged field care, with particular emphasis on ultraportable devices. This review was conducted in accordance with the Scale for the Assessment of Narrative Review Articles (SANRA) guidelines [12], and the literature search followed the PRISMA-trAIce checklist for AI-augmented searches [13]. Records were identified primarily through an AI-assisted literature-discovery tool, the Undermind Deep-Search platform (Undermind Inc.), which interrogates the Semantic Scholar corpus [14]. The search was conducted on 15 June 2025 using a structured query spanning three conceptual domains: (1) ultrasound modalities, (2) disaster and austere settings, and (3) clinical or operational impact, and reached an internal search convergence above 90%. No restrictions on study design, language, or publication date were applied. The tool returned a relevance-ranked set of candidate references; its principal contribution was to surface non-indexed operational, military, and gray-literature reports that conventional database queries capture less reliably. A separate, independently quantified structured multi-database search (for example, PubMed or Embase) was not performed. Inclusion criteria were as follows: (1) original human research data or clinical guidance documents; (2) ultrasound use in disaster, austere, or prehospital conditions; (3) at least one of the four target domains; (4) diagnostic performance, clinical impact, or operational feasibility. Exclusion criteria comprised simulation-only studies without human validation, animal or cadaveric research, urban in-hospital studies outside austere conditions, editorials without original evidence, and duplicative publications. The first author (F.A.) screened all titles, abstracts, and full texts against these criteria, and the search parameters were independently verified by the senior author (A.B.). Of 71 records identified, 63 articles met the inclusion criteria: 32 original studies (prospective *n* = 8; retrospective *n* = 16; case series *n* = 4; case reports *n* = 4), ten systematic, scoping, or meta-analytic reviews, 20 narrative or operational papers, and one guideline. No randomized controlled trials were found. Title, abstract, and full-text screening and final inclusion were performed by a single reviewer (F.A.), a methodological limitation discussed below. The study-selection process is summarized in a flow diagram (Supplementary Fig. 1).

### CBRN risks

CBRN risk refers to scenarios involving chemical, biological, radiological, or nuclear agents [15]. In the context of nuclear, radiological, and biological (NRB) threats, ultrasound holds minimal diagnostic utility, as the rapid identification of the toxic or infectious agent responsible for the exposure is the primary clinical imperative [16]. However, in environments where the risk of healthcare provider contamination is highly likely, traditional tools such as stethoscopes may serve as vectors for pathogen transmission [17–20]. Ultrasonography offers a noninvasive alternative that minimizes physical contact, thereby reducing the potential for cross-contamination between patients and healthcare workers. This benefit was highlighted during the SARS-CoV-2 pandemic, during which many emergency departments (EDs) implemented the use of ultraportable ultrasound devices to triage patients at the point of entry, replacing stethoscope-based pulmonary auscultation [21–23]. However, similar to stethoscope usage, well-defined disinfection protocols must be applied to ultrasound probes to minimize infection transmission [24, 25].

**Fig. 1:**
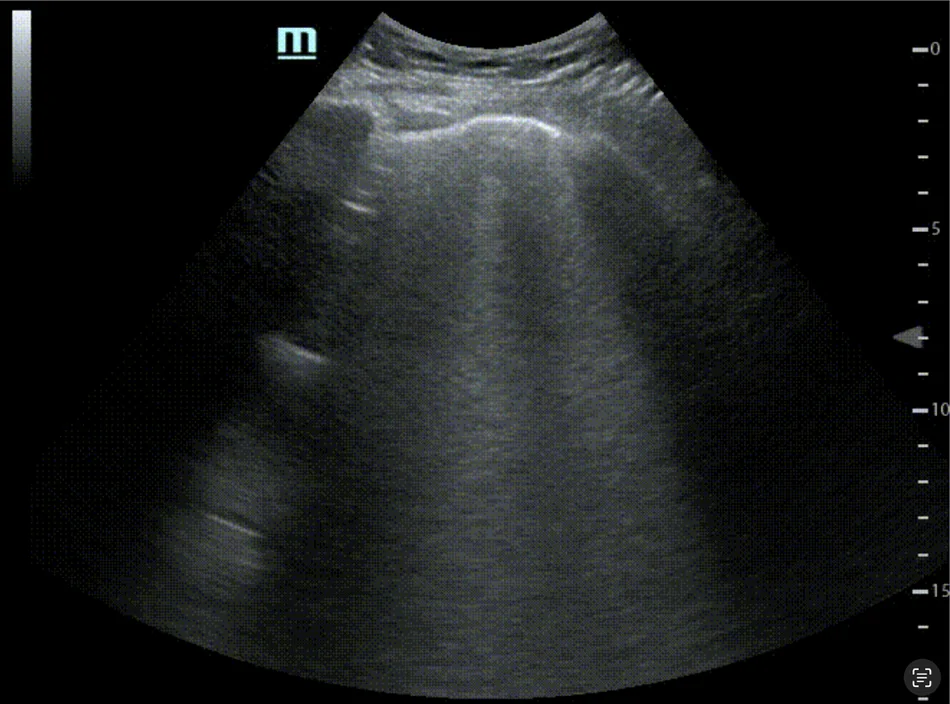
B-lines on lung ultrasound. Vertical hyperechoic artifacts (arrows) originating from the pleural line and extending to the bottom of the screen without fading, indicating alveolar-interstitial syndrome consistent with pulmonary edema. This pattern is characteristic of chemical inhalation injury, acute respiratory distress syndrome, and COVID-19 pneumonia

Disruption of the alveolar-capillary barrier is observed in certain CBRN pathologies, such as chlorine gas poisoning. This disruption leads to the extravasation of inflammatory fluid into the alveoli and interlobular septa, resulting in acute pulmonary edema [26, 27]. Lung ultrasound enables real-time visualization of this process through the detection of B-lines, which are vertical, hyperechoic artifacts that originate from the pleural line and extend to the bottom of the screen without fading (Fig. 1) [28]. Lung ultrasound provides evidence supporting pulmonary edema during acute respiratory distress [29–31]. This is particularly relevant in cases of irritant intoxication, where the onset kinetics of pulmonary pathological lesions and signs of respiratory distress may be delayed [32]. Furthermore, although current evidence remains relatively limited, lung ultrasound may reduce the risk of operator contamination during auscultation and minimize contamination during clinical examinations [33].

Beyond diagnosis, B-line distribution correlates with parenchymal injury extent and enables monitoring of treatment response in CBRN-exposed patients [34–37]. The technique is rapid (approximately 2 min per examination) with adequate proficiency achieved after approximately 10 supervised scans [38].

#### Lessons from COVID-19 as a biological disaster model

The COVID-19 pandemic provided a large-scale, real-world experience with portable ultrasound deployment during a biological emergency. Lung ultrasound demonstrated sensitivities of 90–97% for COVID-19 pneumonia, compared to 60–69% for chest radiography, with diagnostic performance approaching that of chest CT (reported sensitivity of 86–98% across studies) [39, 40]. Critically, lung ultrasound offered several operational advantages directly relevant to disaster settings: absence of ionizing radiation, true bedside availability precluding patient transport through contaminated corridors, repeatability for monitoring treatment response, and reduced healthcare worker exposure by eliminating the need to transfer infectious patients to radiology departments [33]. Multiple centers reported that systematic integration of lung ultrasound protocols reduced chest radiograph and CT utilization by 30–50% during COVID-19 surge periods, yielding resource-efficiency gains essential during capacity-constrained biological emergencies [39, 40]. This pandemic experience supported the feasibility and clinical relevance of ultraportable POCUS as a frontline diagnostic modality during large-scale biological hazard events, with particular value in healthcare systems lacking robust CT infrastructure. Importantly, most of this evidence was generated in controlled hospital and emergency-department settings during pandemic surge periods; its direct extrapolation to open-field biological-disaster scenarios, where image acquisition, decontamination, and operator protection are far more constrained, remains an assumption that has not been prospectively validated and should be interpreted with caution.

### Explosive and ballistic risks

**Fig. 2:**
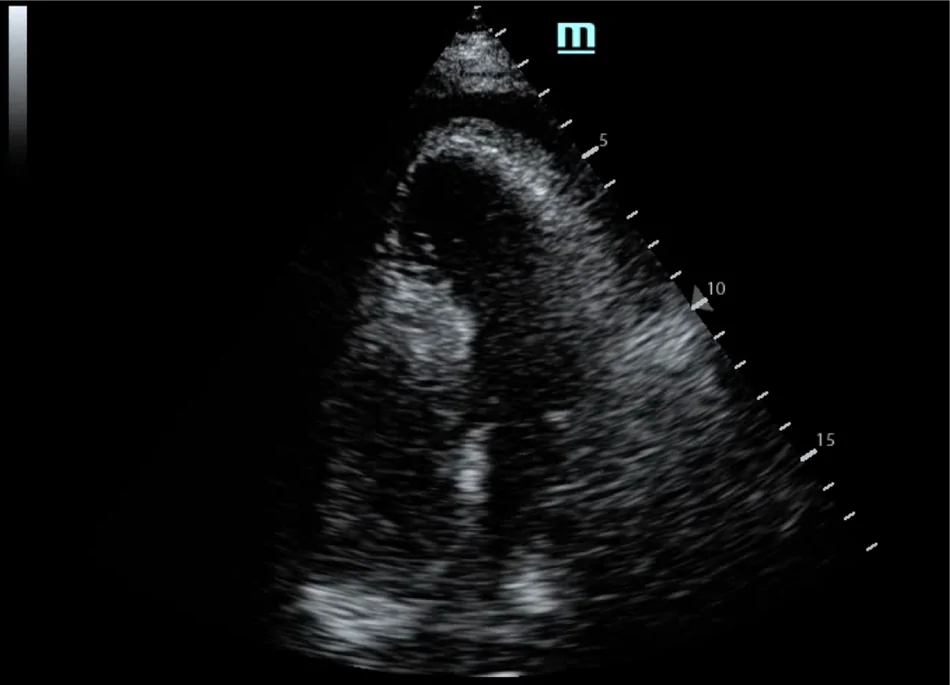
Pericardial effusion on bedside ultrasound. Anechoic fluid collection (arrows) surrounding the heart in a subxiphoid four-chamber view. This finding in the context of explosive or ballistic trauma warrants immediate evaluation for cardiac tamponade

**Fig. 3:**
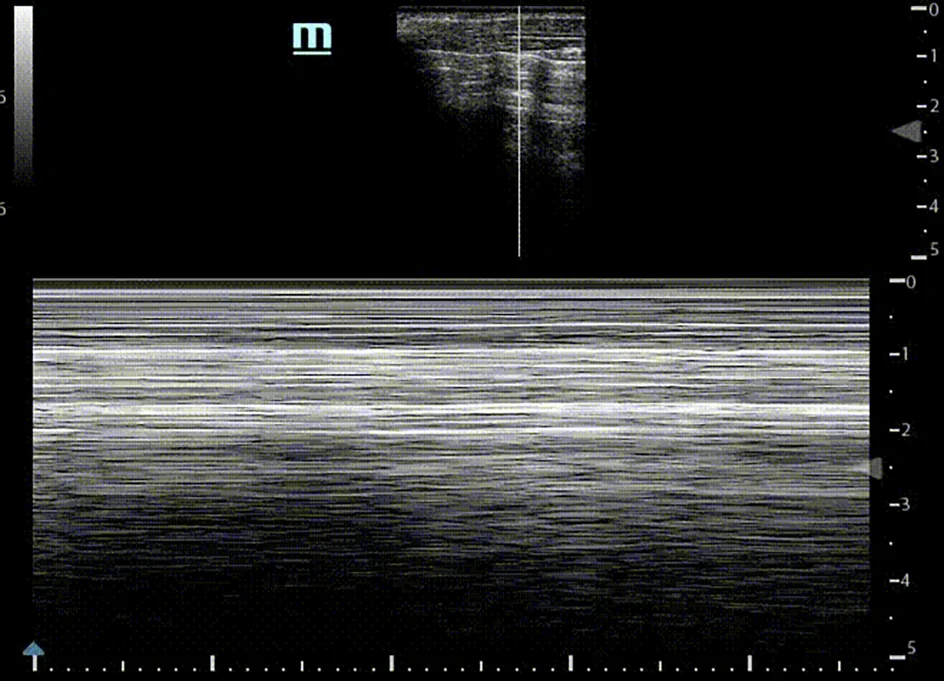
Pneumothorax detection on lung ultrasound. Absence of lung sliding at the pleural interface “barcode sign” on M-mode, indicating pneumothorax. This finding is critical in blast-related injuries, where tension pneumothorax requires immediate decompression

**Fig. 4:**
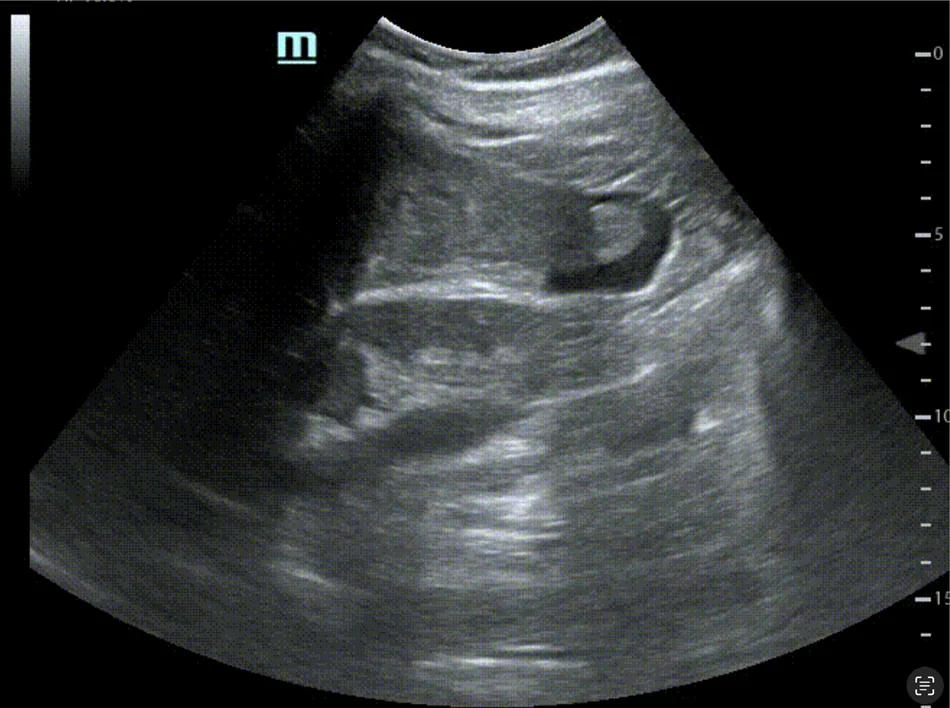
Intra-abdominal effusion in the hepatorenal space (Morison’s pouch). Anechoic free fluid (arrows) between the liver and right kidney on a right upper quadrant FAST view, indicating hemoperitoneum. A positive FAST in an unstable patient in disaster settings triggers damage-control surgery pathways

Explosive and ballistic injuries typically produce penetrating thoraco-abdominal trauma, hemorrhagic shock, and blast-related pneumothoraces requiring immediate identification at the point of care [41]. In these scenarios, ultrasound enables bedside detection of hemoperitoneum, hemothorax, pericardial effusion, and pneumothorax within minutes of patient contact, directly guiding surgical and procedural decisions [42, 43]. In disaster-medicine settings, focused assessment with sonography for trauma (FAST) has demonstrated excellent reliability. A recent meta-analysis including 4263 casualties from earthquakes and armed conflict reported a pooled sensitivity of 92.1% (95% confidence interval (CI): 87.8–95.6) and a specificity of 98.7% (95% CI: 96.0–99.9) for detecting intra-abdominal hemorrhage, with a negative predictive value of 98.9% [44]. Ultrasound enables prompt identification of critical conditions such as pneumothorax, hemothorax, and pericardial effusion (Figs. 2 and 3), all of which have direct therapeutic implications [45]. The detection of intra-abdominal free fluid via FAST (Fig. 4) facilitates early “damage-control” surgical interventions that are closely linked to improved survival outcomes [46, 47]. Notably, delayed diagnosis in such contexts often stems from the poor sensitivity and specificity of clinical signs and hemodynamic parameters [48].

The extended FAST (eFAST) triggers life-saving interventions before computed tomography (CT) is performed. In a multicenter cohort study involving 510 civilian blunt-trauma patients, systematic eFAST led to at least one immediate therapeutic intervention in 54% of cases [47]. In a large series of Helicopter Emergency Medical Services (HEMS) missions (*n* = 6126), ultrasound was employed in 29.6% of dispatches and led to a therapeutic intervention or change in patient destination in 30.9% of scanned cases [49]. During mass-casualty events involving ballistic or explosive mechanisms, eFAST ultrasound proved effective in the triage phase by identifying patients who required urgent surgical care [50–52]. During the 2011 attacks in Norway, nearly all patients arriving at Oslo emergency department underwent eFAST within the first 10 min, enabling the rapid establishment of a streamlined care pathway for damage-control surgery [51]. By aiding triage and facilitating care coordination, eFAST reduces reliance on abdominal CT, an expensive and often limited resource in disaster settings [52].

Military experience further corroborates the utility of prehospital ultrasound. During the Second Lebanon War, prehospital FAST examinations redirected every unstable patient straight to laparotomy and spared 27% of patients further imaging or surgery, effectively conserving surgical and radiological resources in a mass-casualty environment [53, 54]. Field reports from U.S. military operations have demonstrated that handheld ultrasound reduced the frequency of non-indicated thoracostomies and facilitated faster aeromedical evacuations [55].

### Natural disasters

In the aftermath of natural disasters such as earthquakes, floods, tsunamis, and hurricanes, healthcare systems are frequently overwhelmed, while traditional medical infrastructures may be damaged or inaccessible [56]. These crises often result in a sudden and massive influx of patients presenting with diverse injuries, necessitating efficient and rapid triage within the remaining operational care facilities [57]. In addition, due to civilian buildings suffering considerable damage, access to electricity becomes challenging, and radiological infrastructures become unusable. Consequently, the use of ultraportable, easily transportable, and battery-operated ultrasound devices becomes particularly advantageous [58, 59].

Ultrasound triage has been deployed during earthquakes in China, Turkey, Japan, Haiti, and Armenia, primarily using eFAST to identify patients requiring damage-control surgery [42, 57, 60–65]. In the largest reported series, 1386 ultrasound examinations were performed on 1207 patients after China’s 2008 Wenchuan earthquake, with zero false-positive scans and approximately 5% false negatives, detecting hemoperitoneum and hemothorax cases missed by clinical examination alone [57]. Similarly, during the 1988 Armenian earthquake, bedside ultrasound identified significant internal injuries in 20% of 400 victims, directly guiding 16 emergency surgical interventions [63].

In Turkey, Doppler ultrasonography assessed the renal resistive index in crush-injury patients to monitor renal hemodynamics [65]. Japanese post-earthquake responses further demonstrated ultrasound’s role in acute triage and deep-vein thrombosis screening among immobilized survivors [66].

However, it is important to note that the available evidence is heavily concentrated in earthquake response. Prospective data specifically evaluating ultraportable ultrasound during flood, hurricane, or tsunami events remain limited, representing a significant evidence gap.

### Tele-ultrasound and artificial intelligence

A promising development in disaster medicine is the emergence of tele-ultrasonography. Technological advances have enabled the real-time transmission of eFAST ultrasound images via satellite or mobile networks [67, 68]. A study demonstrated successful transmission of handheld FAST video clips from a simulated disaster site over a low-bandwidth (approximately 64 kb/s) mobile network to emergency physicians located 170 miles away, with excellent diagnostic concordance [69]. This approach extends diagnostic capability beyond the local operator’s expertise, enabling less experienced personnel to perform ultrasound under real-time remote guidance from specialists.

Concurrently, the integration of artificial intelligence (AI) into portable ultrasound systems is automating selected components of image acquisition and triage, although the supporting evidence remains preliminary. A single 2023 narrative review described robotic-arm prototypes reported to perform a 30-s automated scan and to classify simulated mass-casualty torso injuries with 86–92% accuracy; these figures derive from proof-of-concept and simulation work rather than field-validated studies and should therefore be regarded as exploratory. The same narrative review cited post-earthquake experience from the 2010 Haiti response in which AI-assisted handheld ultrasound was reported to alter clinical decisions in 70% of 103 cases [70]. However, because this estimate originates from a single secondary source rather than primary, peer-reviewed field data, it should be interpreted with caution. Similarly, a 2024 scoping review of 324 POCUS studies in austere environments reported that ~ 20% of recent deployments incorporated either real-time tele-mentorship or embedded AI algorithms, for example, automated probe positioning or pneumothorax detection. As scoping reviews aggregate heterogeneous and frequently descriptive evidence, this proportion should be viewed as an indicative summary of a rapidly evolving field rather than a precise, primary-data estimate of deployment-ready capability. More broadly, a clear distinction must be drawn between technologies that are promising in experimental or controlled settings and those genuinely validated for deployment in austere or disaster environments; currently, most AI and robotic ultrasound applications remain in the former category.

Even in extraterrestrial settings such as the International Space Station, non-medical personnel have successfully captured diagnostic ultrasound images under remote guidance, highlighting the feasibility of tele-echography in extreme isolation [71, 72]. These findings underscore the potential of combining tele-guidance with ultraportable devices to significantly expand the pool of personnel capable of performing diagnostic ultrasound in disaster settings.

However, AI-assisted ultrasound interpretation remains largely at the proof-of-concept stage; prospective deployment in actual disaster settings has not been fully validated. Reliance on AI without human expert supervision introduces risks related to undetected artifacts, anatomic variants, and algorithm performance in field conditions differing from training datasets.

### Prolonged field care and post-acute management

In resource-constrained settings, ultrasonography becomes particularly valuable during ongoing medical follow-up, as patients may experience clinical deterioration or therapy-related complications after the initial triage and stabilization phase. This section addresses the role of ultrasound in the prolonged or delayed phase of disaster care, when evacuation is delayed and resources remain limited.

#### Vascular access

**Fig. 5:**
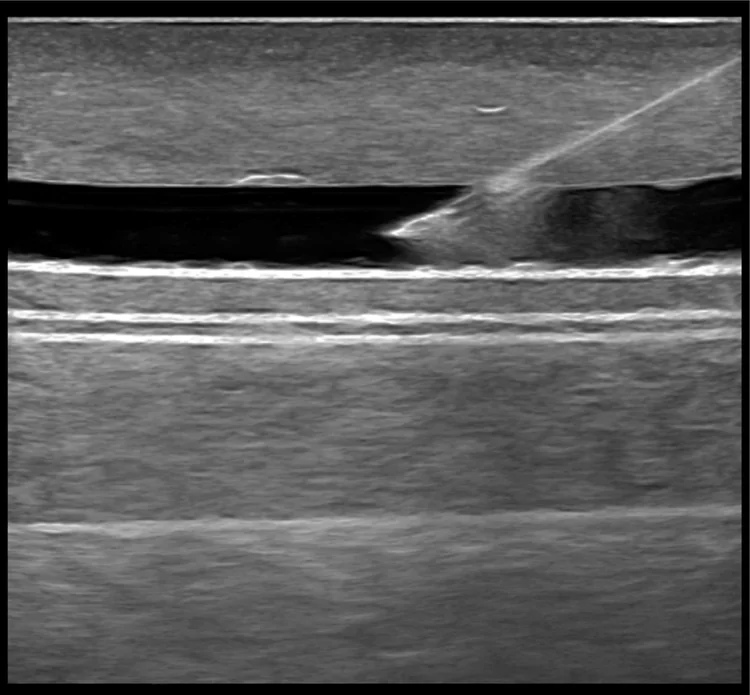
Peripheral venous line placement under ultrasound guidance. Example of a long-axis approach on a low-fidelity simulator. The needle tip (arrow) is visualized entering the target vein, enabling real-time guidance in patients with difficult vascular access due to hypovolemia or austere conditions

Ultrasound-guided peripheral venous access is a critical intervention when visual or tactile guidance is inadequate, such as in patients with hemorrhagic shock, hypovolemia, or during nighttime evacuations without lighting (Fig. 5). Ultraportable ultrasound devices significantly improve cannulation outcomes, raising first-pass success rates from 63% to 77% and achieving 74–95% success in difficult-access cases [73, 74]. Experienced operators report 90% first-pass rates in hypovolemic patients [75], while field reports confirm that battery-powered probes remain effective during nocturnal evacuations where anatomical landmarks are obscured [76]. In the context of disasters, ultrasound-guided procedures were extensively reported during the Wenchuan earthquake [57].

#### Airway confirmation

Confirming orotracheal tube placement is crucial. The incidence of esophageal intubation is estimated at 1% in hospitals [77] and may rise to 6% in prehospital settings where intubations are often more complicated [78]. In emergencies, physicians have limited tools to verify correct tube placement. Ultrasound visualization of the “double-tract sign” showing reverberation artifacts in the trachea and esophagus indicates esophageal intubation, while a single artifact indicates tracheal intubation, enabling rapid correction of tube misplacement.

#### Pain management

Loco-regional anesthesia (LRA) provides effective field analgesia for fractures, dislocations, and complex wounds [79, 80]. Field data from military conflicts, notably the Iraq and Afghanistan wars, highlight LRA’s impact on reducing morphine requirements [81, 82]. By improving analgesic adequacy during evacuation, LRA has also been linked to reductions in chronic pain and post-traumatic stress disorder among wounded soldiers [81].

#### Thromboembolic screening

Evidence indicates that bedside ultrasonography effectively detects venous thromboembolism after disasters, when immobility, dehydration, injury, and limited diagnostic resources increase deep-vein thrombosis (DVT) risk [83]. In Japan, portable scanners identified abnormal venous flow or DVT in five of 11 crush-injury patients after the 2015 Joso flood and 2016 Kumamoto earthquake; angiography confirmed these findings and guided treatment [84]. Surveillance after the Great East Japan Earthquake revealed silent thrombi in high-risk residents, enabling timely anticoagulation [66].

#### Blast-related ocular trauma

Within the “SAFE MARCHE RYAN” algorithm for management of combat casualties, blast-related ocular trauma may necessitate special evacuation and management. Portable ultrasound can rapidly assess globe integrity by demonstrating an intact vitreous chamber outline, aiding triage decisions regarding evacuation urgency. However, conditions such as retinal detachment, while diagnosable on ultrasound [85], represent a lower clinical priority in the immediate disaster context, as they do not require emergent field intervention and are optimally managed in specialized ophthalmology settings.

## Learning curve

The learning curve associated with ultraportable ultrasound offers a notable advantage for disaster preparedness. Unlike traditional diagnostic ultrasonography, which requires 5 or more years of radiology residency with supervision of thousands of individual examinations, or cardiology echocardiography fellowship adding 2–3 additional years of specialized training, disaster-context ultrasound protocols target protocol-driven, binary diagnostic decisions (free fluid present/absent, pneumothorax present/absent, cardiac activity present/absent), achievable with substantially less training.

The European Federation of Societies for Ultrasound in Medicine and Biology and the American Institute of Ultrasound in Medicine have published competency frameworks for emergency ultrasound. EFSUMB Level 1 emergency ultrasound competency requires approximately 150–300 supervised examinations [1]. However, this standard derives from comprehensive emergency ultrasound training encompassing abdominal, vascular, and obstetric applications. Disaster-focused FAST/eFAST training, confined to binary assessment protocols, achieves comparable diagnostic accuracy with fewer total examinations.

Achieving sufficient skill in lung ultrasound, essential for identifying conditions such as pulmonary edema, can often be accomplished after about 10 supervised scans [38]. Similarly, targeted training can swiftly equip non-physician practitioners with the ability to perform FAST/eFAST with clinically acceptable precision [86]. A concise 4-h course enabled Army medics with no prior ultrasound experience to achieve eFAST proficiency comparable to that of emergency physicians (post-training knowledge, approximately 83% compared to 27% at baseline; OSCE performance, approximately 20.8 out of 22 points with no significant difference from emergency medicine residents), although novice medics initially required more time per examination (approximately 9 min vs. 4 min) [87].

Targeted training sessions, even when brief, have resulted in high competency in eFAST usage. Following a 5-min cardiac ultrasound tutorial, 92% of medics were able to obtain an adequate subxiphoid view to evaluate cardiac activity, and a 25-min lung ultrasound session enabled flight paramedics to identify the sonographic lung-sliding sign with approximately 97% sensitivity and > 93% specificity, with 100% accuracy maintained at a 9-month follow-up [88, 89]. In field trauma care, FAST conducted by newly trained providers consistently demonstrates high specificity (approximately 95–100%) with moderate sensitivity, which improves with experience (for example, sensitivity for detecting free fluid may be approximately 61% after a 4-h EMT training, increasing toward 90% as proficiency develops) [86].

Moreover, it has been shown that individuals without medical training can successfully obtain diagnostic ultrasound images, even in highly isolated environments like the International Space Station, often with the help of remote guidance [90]. This underscores the potential of rapid skill transfer and effective use of ultraportable ultrasound devices by a wide range of responders.

This distinction between focused competency-based training and specialist-level expertise is fundamental to understanding the learning curve in disaster medicine. The aim is not to produce radiologist-level diagnosticians in hours but rather to enable protocol-driven assessments that answer specific binary clinical questions with acceptable accuracy. With adequate training, medics and paramedics can perform ultrasonography in disaster situations with diagnostic accuracy close to that of physicians. However, authors consistently emphasize the need for ongoing practice and regular refresher courses to maintain these skills over time, as proficiency declines without sustained exposure.

### Technical specifications and operational considerations

Operational characteristics of ultraportable devices directly impact disaster deployment feasibility. This section addresses technical parameters identified as critical for field implementation.

#### Device classification

Ultraportable ultrasound devices range from probe-only systems (handheld wireless devices with integrated processing, displaying images on paired smartphones or tablets) to tablet-based platforms (standalone tablets with external wireless probes) to cart-based portable systems. The choice of device class involves trade-offs between portability, diagnostic capability, and cost [6, 10].

#### Battery autonomy

Probe-only handheld devices typically operate for 1.5–6 h on rechargeable lithium-ion batteries; tablet-based systems provide 45 min to 3 h for the tablet component (with separate probe charging); cart-based portable systems rely on batteries providing 2–4 h of continuous operation. In prolonged disaster response, battery depletion requires vehicle-based charging, solar charging arrays, or mechanical (hand-crank) generators. For immediate surge response (first 24–48 h), battery autonomy of 2–6 h typically suffices for triage queue assessment; extended operations require power management strategies.

#### Transducers

Available probes typically include phased-array (cardiac and deep abdominal assessment), linear-array (superficial tissues, vascular assessment), and convex-array (general abdominal imaging) configurations. Newer ultraportable devices employ single broadband probes capable of switching between frequencies (2–5 MHz for deep structures, 5–12 MHz for superficial) without requiring probe exchange, reducing operational complexity in field settings [6, 7].

#### Cost

Handheld probe-only systems cost approximately $2000–$5000; tablet-based portable systems range from $4000 to $10,000; conventional cart-based equipment costs $20,000–$200,000 depending on transducer options and software functionality [6, 10]. For disaster response organizations in resource-limited settings, handheld systems below $5000 offer a cost-effective entry point, while developed-nation organizations may justify cart-based systems for comprehensive diagnostic capability.

#### Ruggedization

Probe durability in austere conditions varies with environmental exposure, including salt water (maritime disasters), dust (earthquakes), or chemical residue (CBRN incidents). Ingress Protection (IP) ratings range from IP65 (basic dust and water resistance) to IP68 (submersible) for specialized systems. Some devices comply with Military Standard (MIL-STD) certifications for electromagnetic interference resilience, shock resistance, and environmental durability, though field validation under actual disaster conditions remains limited.

#### Limitations compared to conventional systems

Ultraportable devices achieve spatial resolution of 1–2 mm, adequate for focused assessment protocols (detecting free fluid as small as 200–500 mL), and temporal resolution sufficient for basic cardiac assessment. However, spectral Doppler capability, essential for vascular flow quantification and cardiac hemodynamics, remains limited or absent on handheld systems, constraining detailed hemodynamic evaluation [10]. Screen sizes of probe-only systems (1.5–2 inches on smartphone-paired devices, up to 7–12 inches on tablets) are smaller than conventional systems, reducing image ergonomics and potentially increasing interpretation difficulty under field conditions. Narrower fields of view in handheld systems necessitate more probe repositioning to complete comprehensive examinations, increasing examination time. Image storage and Digital Imaging and Communications in Medicine (DICOM) compatibility challenges persist, as many ultraportable systems use proprietary formats that impede data transfer for remote consultation or medical records [10]. Beyond image quality, this interoperability gap carries important medico-legal and continuity-of-care implications: in austere responses, patients are frequently evacuated to definitive-care facilities, and the inability to export studies in a readable, DICOM-compatible format may impair documentation, downstream clinical decision-making, comparison with subsequent imaging, and medico-legal traceability. Where feasible, devices supporting standardized DICOM export and structured image transfer should be prioritized to preserve continuity of care across the evacuation chain.

## Limitations

Several disaster-specific limitations constrain current evidence and implementation feasibility.

### Operator dependency in austere environments

Performance degradation is expected in high-noise, low-light, high-stress conditions with casualty instability, hypothermia, or altered consciousness complicating probe contact and patient positioning. Most published FAST/eFAST sensitivity and specificity data derive from controlled settings or military training contexts; prospective performance data in actual chaotic disaster scenes remain sparse.

### Geographic and disaster-type bias

Available evidence is heavily concentrated in earthquake responses, particularly in China, Turkey, Japan, Haiti, and Armenia. Evidence for ultrasound utility in CBRN scenarios, floods, and hurricanes remains minimal, limiting the generalizability of findings across the full spectrum of disaster types. Different disaster environments impose distinct technical demands that remain poorly studied: salt water exposure during tsunamis and maritime events, extreme humidity during floods, dust and high ambient temperatures in arid settings, and chemical residue during CBRN incidents may each degrade probe durability and image quality and dictate the Ingress Protection (IP) ratings required for reliable operation. The performance of ultraportable devices under these specific conditions has not been systematically validated, compounding the geographic concentration of the evidence.

### Selection and publication bias

Positive trials demonstrating ultrasound clinical utility are more likely to be reported than operational challenges or negative findings, potentially overestimating the modality’s real-world effectiveness.

### Evidence quality

No randomized controlled trials were identified. The ethical and logistical constraints of randomizing injured disaster patients to imaging protocols preclude RCT design. Most evidence derives from retrospective case series, prospective cohorts with minimal controls, or operational reports lacking formal comparison arms.

### Device durability

Manufacturer specifications address operating temperature ranges (typically 0–40 °C) and humidity limits, but field validation of device functionality after exposure to salt water, dust storms, extreme heat or cold, and chemical contamination remains limited. Actual failure rates and maintenance requirements in austere disaster settings are poorly documented.

### Standardization deficits

There is an absence of universally agreed disaster ultrasonography protocols, a lack of consensus on competency benchmarks for non-specialist operators, and variability in training standards across organizations. This fragmentation complicates systematic integration of POCUS into disaster preparedness frameworks.

### Limited long-term outcome data

Most studies report diagnostic accuracy (sensitivity/specificity) or immediate operational impact (time to diagnosis, imaging workload reduction). Few document effects on survival, morbidity, disability, or resource utilization outcomes. Establishing causal links between ultrasound deployment and improved patient outcomes in disaster contexts requires prospective data that are currently unavailable.

### Single-reviewer study selection and search design

Consistent with the narrative (rather than systematic) design of this review, records were identified primarily through a single AI-assisted discovery tool with manual verification rather than an independent, structured multi-database search, and title, abstract, and full-text screening were performed by a single author (F.A.), with the search parameters independently verified by the senior author (A.B.) but without duplicate independent screening of individual records. This introduces a potential risk of selection bias and reduces reproducibility, and the included evidence base should be interpreted with this limitation in mind; future reviews would benefit from an independent structured search and dual independent screening with formal adjudication of disagreements.

### Immaturity of emerging-technology evidence

Much of the evidence supporting AI-assisted interpretation, robotic ultrasound, and tele-ultrasound derives from simulation studies, single-center feasibility work, or narrative and scoping reviews rather than prospective field validation. Reported performance figures for these technologies should be regarded as preliminary and proof-of-concept, and their readiness for deployment in austere or disaster environments remains unproven. Overreliance on such tools without expert human supervision risks propagating undetected artifacts and algorithm failures under field conditions that differ from training datasets.

## Conclusion

Ultraportable POCUS has emerged as a major adjunct in disaster medicine, bridging critical diagnostic gaps across the disaster continuum. From early triage of victims of natural catastrophes, blast or ballistic injuries, and CBRN incidents to the delayed care of stabilized patients, handheld ultrasound devices enhance clinical assessment, guide life-saving interventions, and optimize scarce medical resources. Their performance, portability, rapid deployment, and expanding integration with telemedicine and AI platforms extend their utility even into the most austere environments.

Technical specifications (battery autonomy of 45 min to 6 h, costs of $2000–$10,000 for handheld systems, and training achievable in 4–25 h) enable deployment to settings lacking conventional imaging infrastructure. Experience during the COVID-19 pandemic supports a role for portable ultrasound in biological emergencies, with lung ultrasound sensitivity approaching that of CT for pulmonary assessment; as these data were generated predominantly in hospital and emergency-department settings, their extrapolation to open-field biological-disaster scenarios remains to be validated. Earthquake and blast-injury literature has established utility in trauma assessment, with pooled FAST sensitivity exceeding 92%.

Despite these advantages, the evidence supporting their impact on hard outcomes such as survival, morbidity, and resource utilization remains limited and is largely derived from observational data, predominantly from earthquake settings. Standardized disaster ultrasonography protocols and competency benchmarks remain lacking. Future research should prioritize: (1) prospective studies evaluating portable ultrasound performance in actual disaster environments across diverse disaster types; (2) multicenter data collection to establish evidence beyond earthquake contexts; (3) long-term outcome studies documenting effects on survival and morbidity; (4) development of consensus disaster ultrasonography protocols and competency standards; and (5) cost-effectiveness analyses for healthcare systems considering acquisition and training investments.

## Supplementary information

ELECTRONIC SUPPLEMENTARY MATERIAL

## Data Availability

Not applicable. All data analyzed in this narrative review are available in the cited published articles.

## Abbreviations

AI: Artificial intelligence
CBRN: Chemical, biological, radiological, or nuclear
CT: Computed tomography
DVT: Deep-vein thrombosis
eFAST: Extended focused assessment with sonography for trauma
FAST: Focused assessment with sonography for trauma
LRA: Loco-regional anesthesia
POCUS: Point-of-care ultrasonography
SANRA: Scale for the Assessment of Narrative Review Articles

## Declarations

### Guarantor

The scientific guarantor of this publication is Dr. Félix Amiot.

### Statistics and biometry

A.B. provided statistical advice for this manuscript. One of the authors has significant statistical expertise. No complex statistical methods were necessary for this paper.
