# Supplementary material for: Ultrasonography in disaster medicine: a narrative review
Source: Insights Imaging. 2026 Oct 13;17:251. doi: 10.1186/s13244-026-02420-3 (PMC13661852; doi:10.1186/s13244-026-02420-3)
Supplement: Supplementary file 1 [file 13244_2026_2420_MOESM1_ESM.pdf]

Ultrasonography in Disaster Medicine: A Narrative Review

ELECTRONIC SUPPLEMENTARY MATERIAL

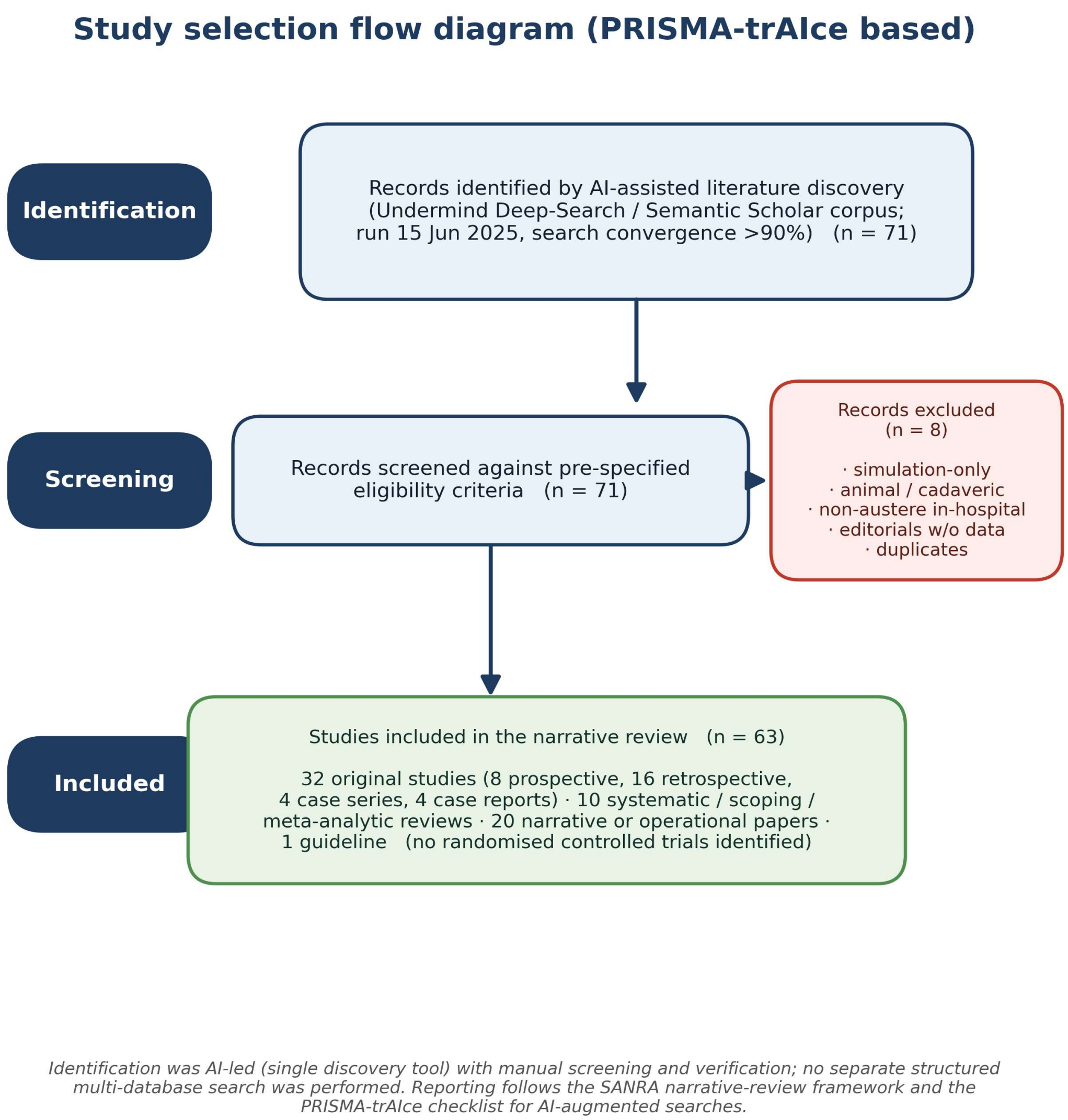

Supplementary Figure 1. Study selection flow diagram (PRISMA-trAIce based). Of 71 records identified through AI-assisted literature discovery (Undermind Deep-Search / Semantic Scholar corpus), 8 were excluded after screening against pre-specified eligibility criteria, yielding 63 included articles (32 original studies, 10 systematic/scoping/meta-analytic reviews, 20 narrative or operational papers, and 1 guideline; no randomized controlled trials identified).
